# The effect of the contract-relax-agonist-contract (CRAC) stretch of hamstrings on range of motion, sprint and agility performance in moderately active males: A randomised control trial

**DOI:** 10.17159/2078-516X/2019/v31i1a6091

**Published:** 2019-01-01

**Authors:** T Burgess, T Vadachalam, K Buchholtz, J Jelsma

**Affiliations:** 1 Division of Physiotherapy, Department of Health and Rehabilitation Sciences, Faculty of Health Sciences, University of Cape Town, Cape Town, South Africa; 2 Division of Exercise Science and Sports Medicine, Department of Human Anatomy, Faculty of Health Sciences, University of Cape Town, Cape Town, South Africa

**Keywords:** sport performance, proprioceptive neuromuscular facilitation, PNF, flexibility, knee

## Abstract

**Background:**

Although stretching is done routinely to prevent injury during explosive sport activities, there is some concern that effective stretching might negatively impact on performance.

**Objective:**

This study’s main objective was to investigate the impact of a specific stretch, the contract-relax-agonist-contract (CRAC) stretch, in which the muscle to be stretched, namely, the hamstrings, is actively contracted and then relaxed. This is followed by the antagonist muscle (the quadriceps) contracting. Secondly, the impact of the stretch on performance was examined.

**Methods:**

A randomised control trial was used. Forty healthy active males between 21 and 35 years old were assigned to either receive three repetitions of CRAC or rest. Hamstring flexibility and the Illinois Agility Test were the primary outcome measures.

**Results:**

The intervention was effective in improving hamstring flexibility by 37% immediately post-application and was maintained for eight minutes thereafter. It had no significant effect on agility or sprint times.

**Conclusion:**

CRAC, when applied to stretch the hamstring muscles of active males, resulted in a large increase of active knee extension range of motion, without decreasing performance. Therefore, CRAC appears to be a safe and effective method of increasing the length of the hamstrings pre-sport activity and should be utilised by sports physiotherapists if deemed necessary. It was also shown to be beneficial following the initial assessment.

Hamstring strains are among the most common sports-related injuries, and account for a significant number of missed competitions for elite athletes in a variety of sporting codes.^[1]^ There is a high rate of hamstring strain re-occurrences which impacts on the athlete’s return-to-play; and indicates the persistent nature of the injury.^[1]^ This is not surprising as, from an anatomical and biomechanical perspective, the hamstring muscle complex is at an increased risk of injury due to its biarticular structure and its functions as a hip extensor and knee flexor.^[2]^ It is particularly vulnerable during sporting activities that involve sudden acceleration/deceleration and jumping, such as field hockey and soccer.^[3]^ Stretching has been advocated as a method of improving or maintaining flexibility and has been used prophylactically in many sporting codes to prevent muscle strains. This is despite the lack of clear evidence for the proposed benefits of improved flexibility in injury reduction.^[4]^ Different stretching techniques, such as static, ballistic and proprioceptive neuromuscular facilitation (PNF), have been used.^[5]^

The concept of PNF stretching is based on neurophysiological principles. The methods include reciprocal and autogenic inhibition through manual contact, diagonal and spiral movement of the limb, and normal timing. These methods facilitate sensory input, functional muscle contraction (including synergist overflow), sequential contraction and coordinated movement, respectively.^[6]^ There are several PNF stretching techniques which may be utilised, including the contract-relax-agonist-contract method (CRAC) which combines different elements of a stretch.^[7]^

In a CRAC stretch, the antagonists (e.g. hamstrings) are first passively stretched, followed by a six to 15 second isometric contraction against resistance at the point of limitation. This contraction is immediately followed by a six to 15 second concentric contraction of the agonists (e.g. quadriceps). This is followed by 20 seconds of rest before the cycle is repeated four to five times. The use of the isometric contraction of the antagonists (hamstrings) at the end of the range serves a dual purpose: the fatigue of fast-twitch fibres and sensory receptor stimulation. Firstly, fatigue of the fast-twitch fibres reduces their capacity for maximum force production when exposed to subsequent stretch resistance.^[3]^ Isometric contraction induces post-isometric relaxation in the muscle, which results in reduced muscle tone. Post-isometric relaxation has been defined as the 15 second refractory period after isometric contraction during which the new point of resistance of a joint or muscle may be achieved more easily.^[8]^

Secondly, sensory receptor stimulation occurs due to the effect of isometric contraction on the Golgi tendon organs (GTO) and muscle spindle fibres. The isometric contraction of a stretched muscle serves to pre-tension the GTO.^[9]^ The increase in tension causes inhibition of the contracting antagonist, while there is simultaneous stimulation of the agonist muscle (quadriceps) by the process of autogenic inhibition.^[9]^ During the transient period of decreased hamstring sensitivity and excitability caused by autogenic inhibition, post-isometric relaxation and H-reflex inhibition, the concentric quadriceps contraction is initiated. Although hamstring electromyographic (EMG) activity may increase, smooth knee joint motion should occur during the quadriceps contraction as the hamstrings are relaxed according to the principle of reciprocal inhibition.^[10]^ The increased stretch tolerance and pain threshold after stretching, combined with the above neurological mechanisms, should facilitate increased knee range of movement (ROM) and hamstring muscle length following hamstring CRAC stretching.

Of concern is that previous studies found that there may be a stretch-induced deficit in muscle performance after an acute stretching protocol.^[4]^ This effect seems more pronounced with a static stretch of longer duration.^[4]^ Furthermore, there is also conflicting evidence for the degree of stretch-induced deficit between different stretch techniques.^[11]^ The stretch-induced performance deficit concept is based on the theory that a more compliant musculotendinous unit has non-optimal sarcomere cross-bridge kinetics after stretching. This delays the production of tension within the sarcomere and the subsequent force transmission from the musculotendinous unit to the tenoperiosteal junction.^[11]^

## Statement of the problem

Despite evidence that CRAC stretching is effective in increasing the length of the hamstrings, there are only a few studies that have investigated both the changes in hamstring flexibility following CRAC stretching and the impact on the function of this increased length.^[12]^ While the duration of maintained flexibility has been defined following acute static and contract-relax (CR) hamstring stretching protocols, this effect has not been established following acute hamstring CRAC stretching.^[13, 14]^ The principle aim of this study was to determine the effects of the CRAC stretch of the hamstrings on functional measures of muscle performance, such as flexibility, agility and sprint performance. The secondary aim was to establish the duration of maintained hamstring flexibility after the acute application of the hamstring CRAC stretch.

## Methods

### Study design

The study used a randomised control experimental design to investigate the short-term effects of a CRAC technique on the hamstring muscles in fit adult men. Participants were randomly assigned to an experimental group that received a CRAC stretch or a control group that received no intervention

### Participants

Participants included healthy, active males (21 to 35 years old) who participated in three and five hours of physical activity per week. They were recruited for the study through advertisements placed at gymnasiums in Cape Town, South Africa, and through word of mouth. Data from a previous study that measured hamstring flexibility using the active knee extension test were used to ensure that the sample size would provide sufficient statistical power.^[14]^ In order to detect the smallest meaningful difference of 15º between the mean range of movement of the two groups with a standard deviation of 10º, with an alpha of .05 and a power of 90% statistical power, 20 participants were required for each group. Forty participants were therefore recruited to participate in the study.

Participants were required to complete a questionnaire that requested relevant medical, surgical and training-related history as a method of screening for possible exclusion criteria and to determine participant eligibility for the study. Participants that had a previous history of hamstring injury or pathology of the hips, knees, thighs or lower back over the last three months; regular use of muscle relaxants, analgesic, steroidal or non-steroidal anti-inflammatory drugs; orthopaedic or neuromuscular diseases of the lower limbs were excluded from the study. Participants were randomly assigned to either the experimental group or control group at the baseline testing session, following the completion of the informed consent form and relevant questionnaires. Randomisation was conducted by asking participants to draw a piece of paper from an envelope. The envelope contained an equal number of “experimental” and “control” group slips.

### Instrumentation

Body mass (kg) was documented using a calibrated scale, and stature (m) was recorded using a wall-mounted stadiometer.

Hamstring flexibility was assessed using the active knee extension (AKE) test.^[15]^ Participants were placed in a supine position, without the use of a pillow. They were also instructed to allow the ankle to plantar flex during testing to limit the effect of potential increased neural tension that may occur if the ankle went into dorsiflexion. An adjustable strap was placed over the anterior superior iliac spine to limit pelvic movement during testing. An additional strap was placed over the thigh of the leg not being tested to maintain hip extension. The leg being tested was placed on a wooden platform which was used to maintain 90º of hip and knee flexion. These positions were established using a universal goniometer. An inclinometer was aligned with the head of the fibula and lateral malleolus and adjusted to zero. The participants were instructed to extend the knee actively at a slow rate to avoid hamstring muscle spindle excitation until the first onset of a stretch sensation was perceived, as opposed to a feeling of discomfort. At this point, the angle on the inclinometer was recorded. The participant then returned the leg to the starting position and rested for one minute. The average of three inclinometer recordings was recorded. The AKE test was then repeated on the opposite leg.

The Illinois Agility Test was used to determine agility performance.^[16]^ The test involves explosive speed, rapid changes of direction, deceleration and the ability to maintain momentum and balance as a measure of four-directional agility. Previous studies have determined that the Illinois Agility Test is a valid and reliable method of testing agility.^[16]^

Sprint performance was determined by measuring the sprint time over 10 m and 25 m respectively. Participants were given standard verbal encouragement during the test and were requested to complete the course “*as fast as possible*”. Timing for both the agility and sprint tests was manually performed on a stopwatch. The reliability of the 10 m sprint has previously been established.^[17]^

The method described by McAtee ^[8]^ and Schuback et al.^[6]^ was used for the CRAC procedure. In the same position used to test AKE, the primary investigator (a physiotherapist) guided the leg into a straight leg raise until the participant reported the first onset of a stretch sensation in the hamstring muscles. This position was maintained for 15 seconds, with the primary investigator supporting the participant’s ankle by means of this investigator’s shoulder. The participant then performed a maximal isometric contraction of the muscles for six seconds pushing the leg into hip extension. The primary investigator resisted the contraction at the level of the participant’s ankle to assist ergonomic endurance. Immediately after the hamstring muscle’s isometric contraction, the participant was asked to perform a concentric hip flexion muscle contraction against the primary investigator’s manual resistance. The primary investigator encouraged the participant to reach the hip flexion limit and to maintain this for six seconds. A 20 second rest after the concentric hip flexion marked the end of one repetition. Three repetitions of the CRAC stretch were performed for each leg by the primary investigator using standardised verbal instructions to ensure consistency and maximum cooperation from participants.

### Procedure

The study was granted ethical clearance by the University of Cape Town’s Faculty of Health Sciences Human Ethics Research Committee (Ethical clearance no. 200/2009). This study was performed in accordance with the ethical principles outlined in the WMA Declaration of Helsinki, Fortaleza, Brazil, 2013.

Participants were tested individually and required to attend three testing sessions at similar times on alternate days over the course of one week. All tests were conducted on a non-slip indoor track thereby ensuring a consistent testing environment. Body mass and stature were assessed in all participants. Participants were then randomised into either the experimental or control group and familiarised with all testing procedures. The experimental group was also familiarised with the CRAC stretch via a visual demonstration performed by the primary investigator. The participants’ hamstring flexibility, agility and sprint performance were then measured.

During the second session, all participants had their pre-intervention hamstring flexibility recorded. Participants in both groups performed a standardised warm-up which consisted of five minutes of cycling between 100 watts to 120 watts on a stationary cycle ergometer with a magnetic resistance flywheel.

Participants in the experimental group received a CRAC stretch performed bilaterally by the primary investigator. Participants in the control group rested supine for six minutes which was the same duration as for the experimental group’s CRAC stretch. They then had their hamstring flexibility measured using the AKE test. This was followed immediately by recording the better of two trials of agility and sprint performance tests measured using the Illinois Agility Test and Sprint Test, respectively.

At the third testing session, the duration of effect of the CRAC stretch was assessed through repeated measures. For all participants, pre-intervention hamstring flexibility was recorded bilaterally, followed by the standardised warm-up. Participants in the experimental group then received the CRAC stretch, performed on each leg, by the primary investigator. Participants in the control group rested supine for the same duration as was required for the CRAC stretch to be performed.

### Statistical analysis

Statistical analyses were performed using Statistica software [StatSoft, Inc. (2007). STATISTICA (data analysis software system), version 8.0. www.statsoft.com]. Normality was determined using the Kolmorogov-Smirnov test. Differences in the descriptive variables between the experimental and control groups were assessed using an independent t-test. Statistical significance for the two main effects of group and time, and the interaction (group × time) of duration of effect of the CRAC stretch were assessed using a two-way analysis of variance (ANOVA) with repeated measures. Tukey’s HSD post hoc comparisons were performed where necessary. Differences in the three dependent variables (flexibility, agility and speed) were compared at pre- and post-intervention periods between groups using an independent t-test. As the body mass index (BMI) differed between groups, the Pearson’s correlation between BMI and ROM of the right leg and the change in ROM was calculated. All data are presented as the mean ± standard deviation (SD), unless otherwise stated. Statistical significance was accepted as p ≤ 0.05.

## Results

The participants from both groups took part in all the sessions. The descriptive characteristics of all participants are shown in Table 1. There was a significant difference between groups in body mass (p=0.02), with participants in the experimental group having a significantly higher body mass compared to participants in the control group. There were no significant differences between groups for any other descriptive variables.

The difference in hamstring flexibility (measured as degrees of active knee extension) prior to and immediately following CRAC stretching in the experimental group as compared to the control group (no intervention) is shown in Table 2. Note that an increase in AKE indicates increased hamstring flexibility.

There was a significant interaction between groups over time (F^(7, 266)^=38.95; p < 0.001) with an increase in active knee extension angle of the experimental group post-CRAC stretch compared to the control group. Experimental group knee extension angle, immediately post-CRAC intervention, remained significantly increased for the duration of eight minutes compared to that of the experimental and control group’s baseline knee extension angle (p < 0.001).[Fig f1-2078-516x-31-v31i1a6091]

There were no significant correlations between BMI and either baseline ROM of the right leg (r=0.20, p=0.22) or change in ROM (r=−0.21, p=0.20).

## Discussion

The hamstring CRAC intervention was effective in improving hamstring flexibility immediately post-application and maintained significant hamstring flexibility for a duration of eight minutes thereafter. The CRAC intervention had no significant effect on agility or sprint times in moderately active males. The two groups were identical, apart from body weight, but as no correlation was found between BMI and change in ROM, it is likely that any difference in outcome was as a result of the intervention.

The role of CRAC in increasing hamstring length is supported by studies which reported similar results and was not unexpected.^[5, 12]^ It is difficult to compare the increase with other studies as most did not report the percent increase but rather the mean gain in ROM, which ranged from 1.6° to 15.7° after using CRAC on hamstrings.^[18]^ Using the AKE test, an increase of 10% in ROM was reported after using PNF techniques.^[11]^ The 17.7° or 37% increase in range gained by CRAC compares well with the results of similar studies.^[5, 12]^

In addition, the duration of effect was longer than previously reported. Depino et al. reported a maximum duration of effect of three minutes after a static stretch and Spernoga et al. reported significantly improved flexibility up to six minutes after a contract relax stretch.^[13, 14]^ The practical relevance of the duration of hamstring CRAC stretch employed in this study is that three repetitions of the stretch (which would take approximately three minutes in total to perform on a patient) would result in increased flexibility for a minimum time of eight minutes. Optimal timing of performance for warm-up or therapeutic exercises would be within this window of eight minutes after the stretch application, which could take place both pre-match and at intervals.

The possible effects of CRAC on performance were more of a concern. However, the results indicate that CRAC does not seem to have the deleterious effects on performance that have been previously reported, particularly with static stretches.^[4]^ As small decrements in performance can have major implications for elite sportspersons, it is reassuring that CRAC does not result in decreased performance. However, it is recommended that further studies be conducted using electronic timing to ensure greater precision of measurement.

## Conclusion

In conclusion, a set of three repetitions of CRAC applied to stretch the hamstring muscles of active males will result in an expected increase of up to 37% in active knee extension ROM, without decreasing performance. It is suggested that CRAC is a safe and effective method of increasing the length of the hamstrings during pre-sport activities and should be utilised by sports physiotherapists, provided that it is deemed necessary and beneficial following the initial assessment

Future studies should also investigate chronic adaptations following regular long-term hamstring CRAC stretching and examine these effects on sprinting and agility tests in comparison to the effects of acute CRAC applications, such as the intervention used in this study.

## Figures and Tables

**Fig. 1 f1-2078-516x-31-v31i1a6091:**
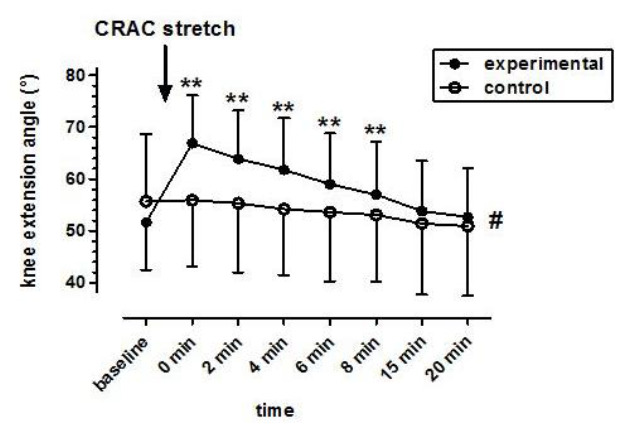
Differences in duration of effect for participants in the experimental group (n=20) and control group (n=20). Data are presented as mean ± standard deviation. Significant differences: ** experimental baseline vs. experimental 0, 2, 4, 6 and 8 min (p < 0.001). # interaction of group × time (p < 0.001).

**Table 1:** Descriptive characteristics of participants in the experimental (n = 20) and control (n = 20) groups

Variable	Experimental	Control
Age (years)	24.1 ± 4.1	24.5 ± 3.9
Stature (m)	1.76 ± 0.06	1.75 ± 0.06
Body mass (kg)	79.0 ± 11.1	72.0 ± 7.5*
Body mass index (BMI)	25.3 ± 3.4	23.4 ± 2.

Data are expressed as mean ± standard deviation.

* indicates p = 0.02

**Table 2:** Comparison of pre- and post-intervention scores between the experimental (n = 20) and control (n = 20) groups

Variable		Experimental	Control	t	p
**Hamstring flexibility (°)**	**Pre-intervention**	48.2 ± 8.5	56.9 ± 13.4	−2.57	0.12
	**Post-intervention**	65.9 ± 8.6	58.0 ± 13.2	2.26	0.03*
**Agility score (s)**	**Pre-intervention**	16.9 ± 0.8	17.1 ± 1.8	−0.44	0.66
	**Post-intervention**	16.4 ± 0.8	16.7 ± 1.6	−0.88	0.38
**Best 10 m sprint (s)**	**Pre-intervention**	2.0 ± 0.1	2.0 ± 0.3	−0.20	0.84
	**Post-intervention**	2.0 ± 0.1	2.0 ± 0.8	0.26	0.80

Data are expressed as mean ± standard deviation.

* indicates p <0.05
